# Supplementary material for: NUDT2 initiates viral RNA degradation by removal of 5′-phosphates
Source: Nat Commun. 2021 Nov 25;12:6918. doi: 10.1038/s41467-021-27239-y (PMC8616924; doi:10.1038/s41467-021-27239-y)
Supplement: Supplementary file 1 — Supplementary Information [file 41467_2021_27239_MOESM1_ESM.pdf]

Supplementary Figure 1

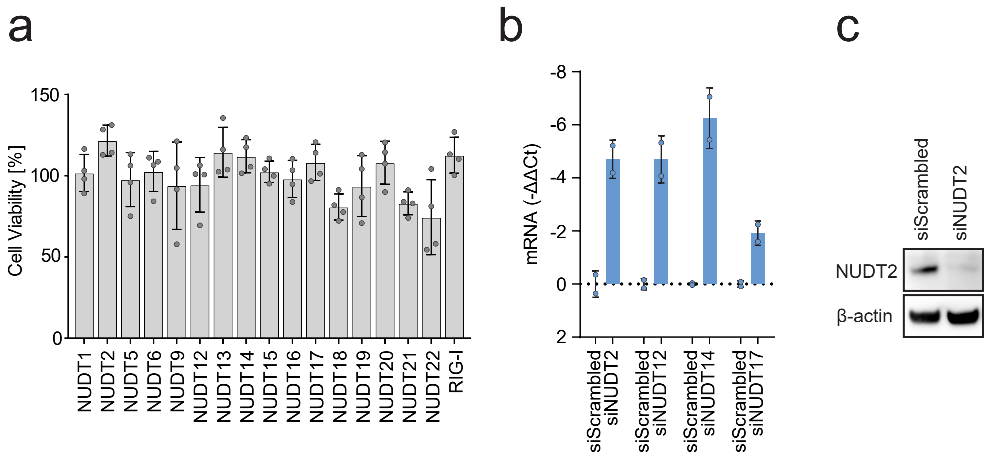

Supplementary Figure 1 | Cell viability after NUDT depletion and confirmation of knockdown. (a) Cell viability after siRNA knockdown. 72 h after siRNA transfection into HeLa cells, cell viability was tested using a cell titer glow assay. Relative cell viability was assessed by assuming the average cell viability of the whole siRNA screen set as 100% viable. The graph shows an average of four independent experiments done in technical duplicates ± SD. **(b)** Cells were treated with siRNA targeting the transcript of *NUDT2*, *NUDT12*, *NUDT14*, *NUDT17*, or control (siScrambled), and mRNA abundance of the respective cellular targets was quantified using RT-qPCR normalized to GAPDH. Bar graphs show the mean fold change of the targeted sequence vs. control ± SD of two independent replicates. **(c)** Expression of NUDT2 in cells transfected with siNUDT2 or control (siScrambled) assessed by western blotting.

Supplementary Figure 2

a

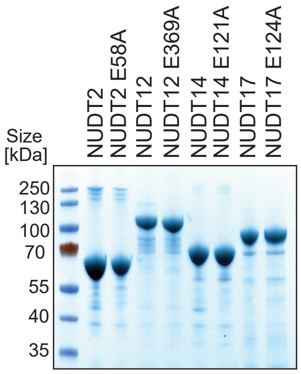

b

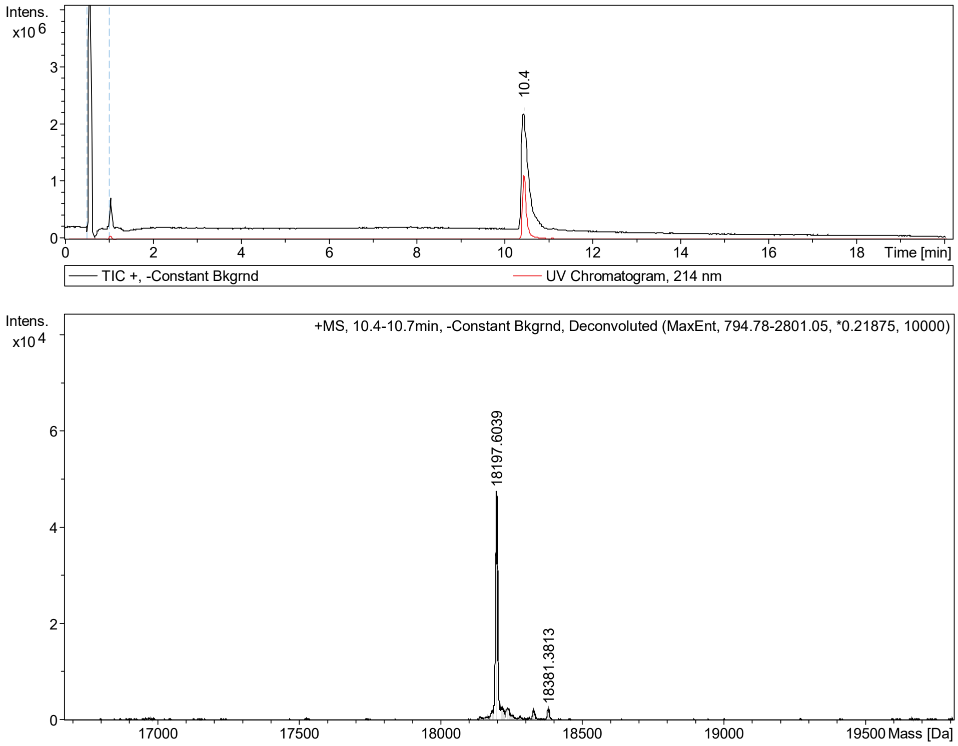

c

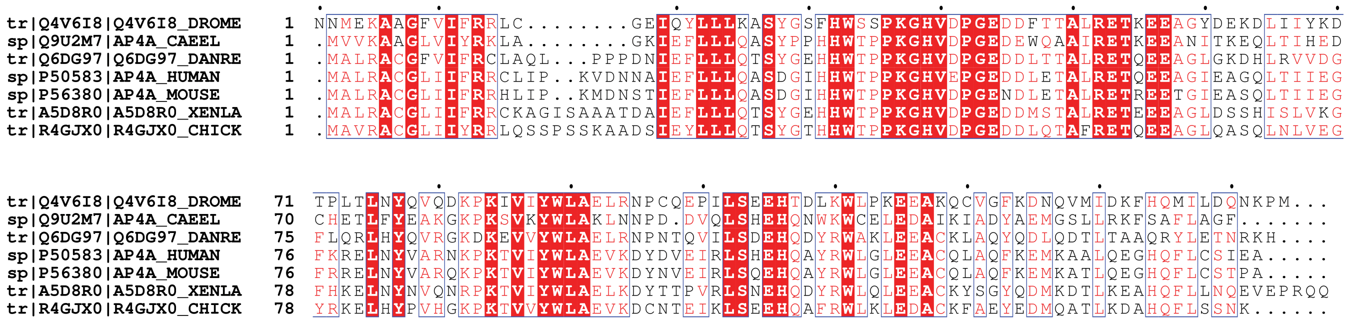

Supplementary Figure 2 | NUDT2 localization and cross-species sequence alignment. (a) Coomassie-stained SDS-PAGE of His6-MBP-tagged recombinant Nudix hydrolases, including mutants bearing point mutations in the catalytic Nudix box (n = 1). **(b)** Total mass determination of NUDT2 by top-down LC-MS analysis, including the total ion current chromatogram (TIC, top) and the extracted ion chromatogram (bottom). **(c)** Sequence alignment of NUDT2 proteins from *Homo sapiens*, *Danio rerio*, *Caenorhabditis elegans*, *Mus musculus*, *Xenopus laevis*, *Gallus gallus*, and *Drosophila melanogaster*. Colors are depicted as following: red box, white character shows a strict identity of residues; red character shows a similarity in a group; a blue frame shows similarities across groups.

Supplementary Figure 3

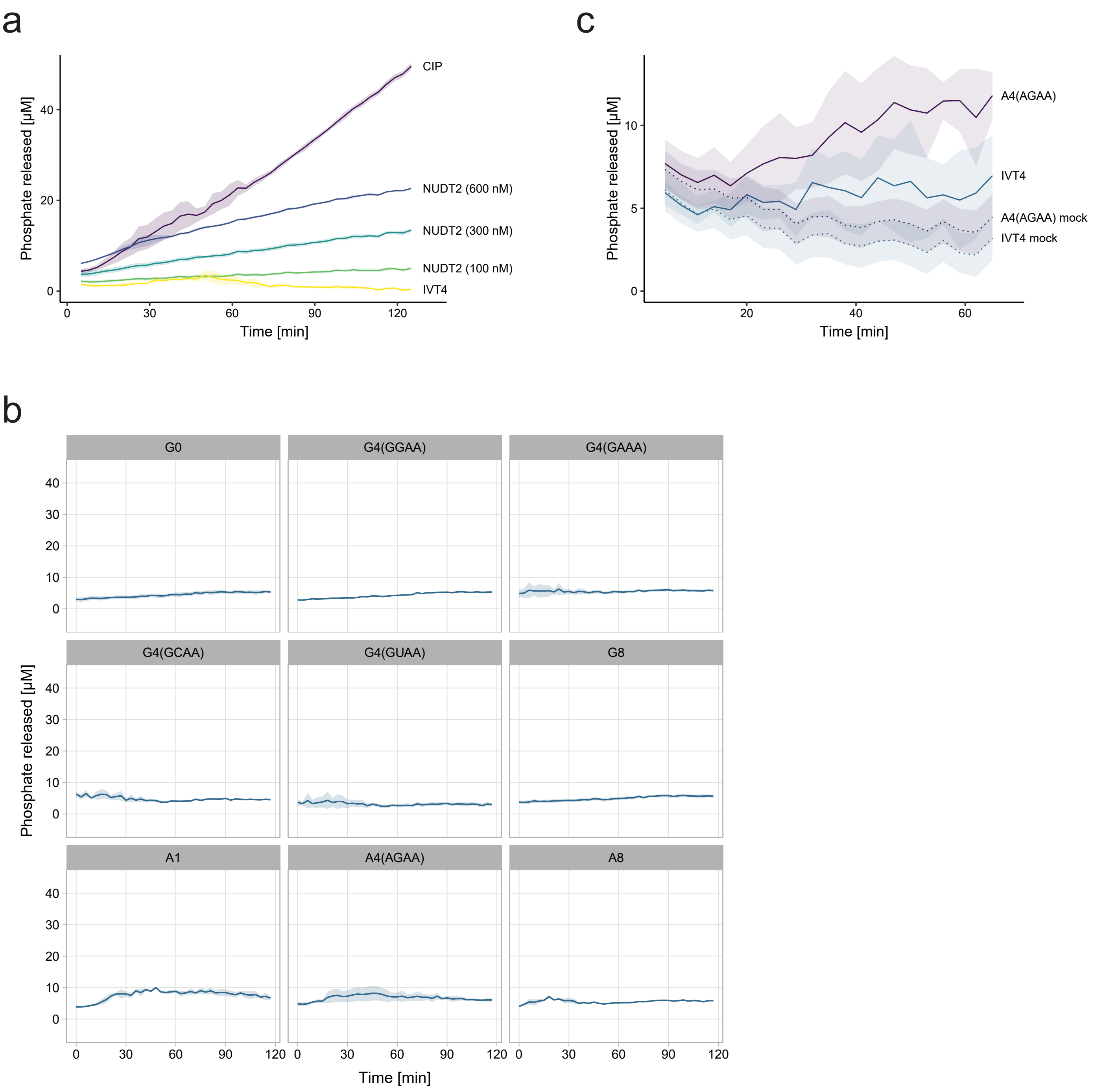

Supplementary Figure 3 | Time-resolved phosphate release assay. (a) Time-resolved EnzChek assay to quantify released phosphate from incubated IVT4 RNA with different concentrations of NUDT2. Spectrophotometric measurements of the samples were performed every 3 min. The line graph shows the mean of three independent experiments with the ribbon indicating ± SD. **(b)** Complementary to Fig. 3c. RNA substrates depicted in Fig. 3b were incubated with NUDT2 E58A mutant at a concentration of 600 nM over a time-course of 2 h utilizing the EnzChek assay to quantify phosphate release. Spectrophotometric measurements were performed every 3 min. The line graph shows the mean of three independent experiments with the ribbon indicating ± SD. **(c)** As (a) but using RppH to dephosphorylate an RNA substrate which is base-paired on its 5'-end (IVT4) and one that has an unpaired 5'-overhang (A4(AGAA)) in comparison to untreated RNA substrates. The line graph shows the mean of three independent experiments with the ribbon indicating ± SD.

Supplementary Figure 4

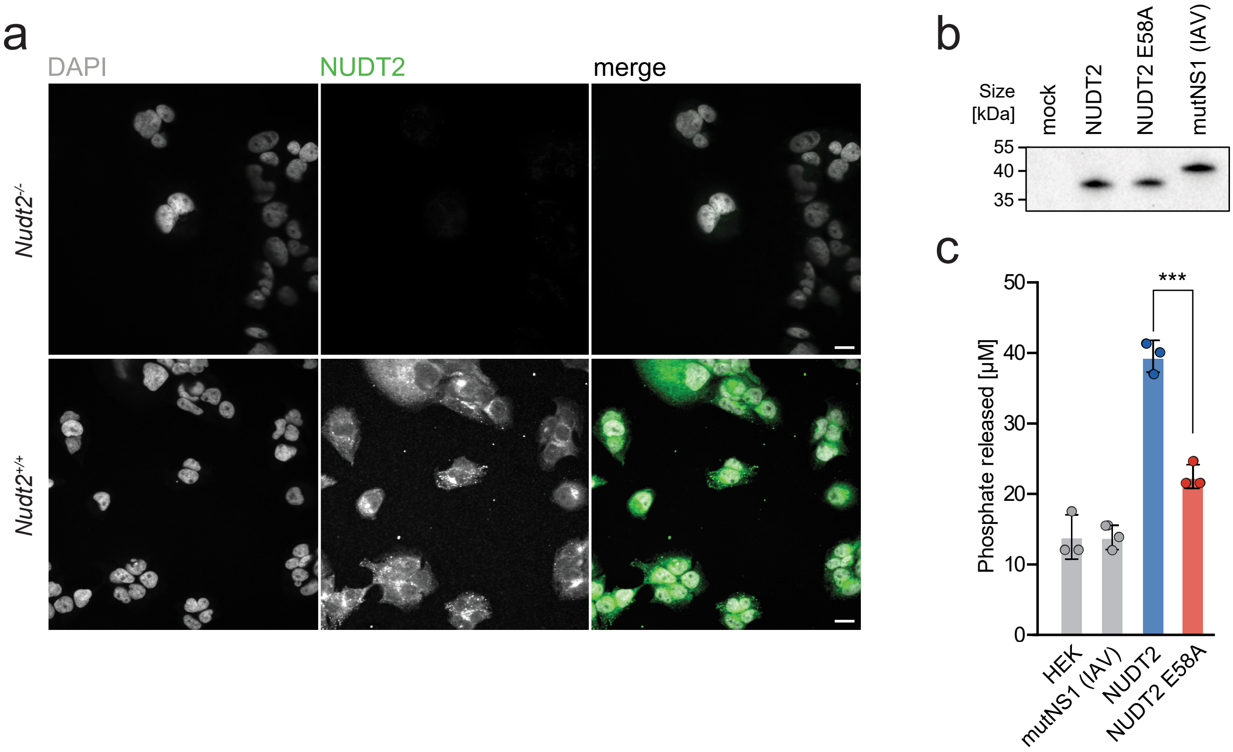

Supplementary Figure 4 | Human NUDT2 subcellular localization and function. (a) Huh7.5 and Huh7.5 deficient for NUDT2 cells were stained for NUDT2 and analyzed by confocal microscopy. Representative images of three biological repeats are shown. The scale bars represent 10 µm. **(b)** HEK293T cells were transfected with plasmids encoding Myc-tagged NUDT2, NUDT2 E58A, and the control mutNS1 (IAV) or left untransfected (mock). Myc-tagged proteins were precipitated, and precipitation efficiency was evaluated by western blotting against Myc. **(c)** Precipitates from (b) were incubated with PPP-RNA (IVT4) for 3 h and phosphate release evaluated using a malachite green assay. Bar graphs show the mean of three independent replicates ± SD, *** $p<0.001$ as analyzed by nonparametric two-tailed t-test.

Supplementary Figure 5

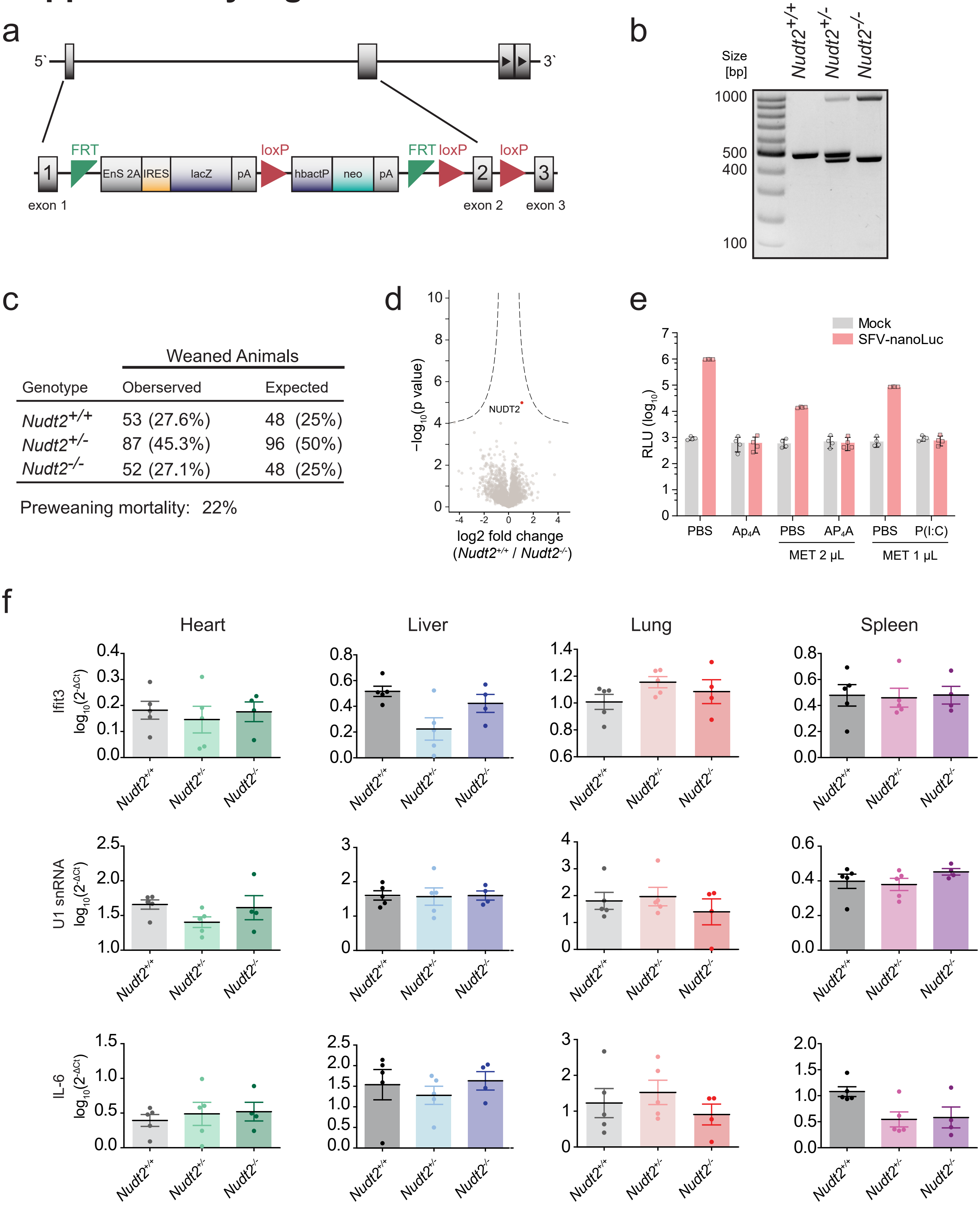

Genotype	Weaned Animals: Oberserved	Expected
Nudt2+/+	53 (27.6%)	48 (25%)
Nudt2+/-	87 (45.3%)	96 (50%)
Nudt2-/-	52 (27.1%)	48 (25%)

Preweaning mortality: 22%

Supplementary Figure 5 | Characterization of NUDT2 deficient mice. (a) Schematic overview of *Nudt2* targeting in mice. Depicted is the *Nudt2* gene locus with a tm1a cassette inserted after the first exon and encoding for the lacZ gene, with a splice acceptor, a polyA site, and a neomycin resistance gene. **(b)** Genotyping PCR of $Nudt2^{tm1a/tm1a}$ ($Nudt2^{-/-}$) mice. PCR amplification results in a wild-type allele detectable at 492 bp, whereas the inserted tm1a cassette is detectable as bands of 417 bp and 912 bp. **(c)** Mendelian ratios were observed from 192 weaned animals originating from $Nudt2^{+/-}$ breeding pairs compared to the expected Mendelian distribution. **(d)** Proteome analysis of bone marrow cells isolated from NUDT2 knockout ($Nudt2^{-/-}$) and control mice. Volcano plots display proteins that were significantly up-or down-regulated in cells lacking NUDT2 (right) or wild-type (left) bone marrows (student´s *t*-test, FDR ≤ 0.05, 6 biological replicates). **(e)** Influence of extracellular applied or intracellular delivered Ap_4A or poly(I:C) on the growth of SFV6-2SG Nano-Luc (SFV) at an MOI of 0.0035. Virus growth was assessed by the measurement of renilla luciferase. The bar plot shows the mean ± SD of four independent replicates. **(f)** Levels of Ifit3, Il6, or U1 snRNA in the heart, liver, lung, or spleen of wild-type mice or $Nudt2^{-/-}$ mice were quantified by RT-qPCR analysis. Data were normalized to murine Tbp RNA. Shown is the mean ± SEM. Every dot represents an individual mouse (n = 5 for $Nudt2^{+/+}$ and $Nudt2^{+/-}$ and n = 4 for $Nudt2^{-/-}$).

Supplementary Figure 6

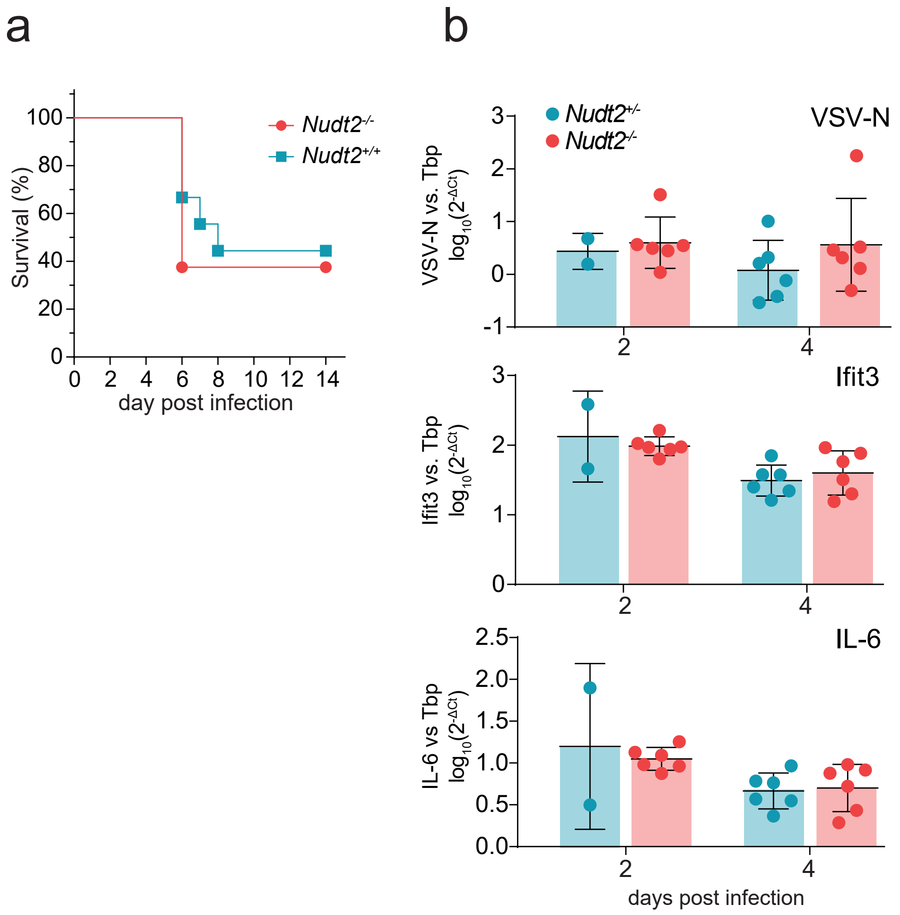

Supplementary Figure 6 | in vivo infection experiments. (a) Survival rates of 8–12 week old wild-type (*Nudt2*$^{+/+}$) (9 animals) or NUDT2 knockout (*Nudt2*$^{-/-}$) mice (8 animals) infected intranasally with 5e6 PFU of VSV in 20 µL PBS. **(b)** Viral RNA load and levels of Ifit3 and Il-6 in the cerebrum of wild-type and knockout mice infected with VSV. RNA levels were quantified by RT-qPCR analysis using specific primers for the N-transcript of VSV. Data were normalized to murine Tbp RNA. Shown is the mean ± SD. Every dot represents an individual infected mouse (n = 2 for NUDT2-/- at 2 days post infection and n = 6 for the other conditions).

Supplementary Table 1

siRNA target sequences used in the siRNA screen

Sequence Name	siRNA target sequence 5’->3’
Hs_NUDT1_3	CTCCTGCTTCAGAAGAAGAAA
Hs_NUDT1_4	CCGGGTTTCATCTGGAATTAA
Hs_NUDT1_5	CCGCGAGGTGGACACGGTCTA
Hs_NUDT2_10	CAGGCATCAGATGGCATTCAT
Hs_NUDT2_7	TGCCAGGGTCCTGCAGTTATA
Hs_NUDT2_8	AGGATCCTTGTGGGCCTTCTA
Hs_NUDT5_2	CAGGCTTGTCAAACTGTACTA
Hs_NUDT5_3	TACATGGATCCTACTGGTAAA
Hs_NUDT5_6	TTCCTACGCTCTAGCACTGAA
Hs_NUDT6_5	CACGCAGAATCGGATTCATCA
Hs_NUDT6_6	CTGGTTGTACAAGATCGAAAT
Hs_NUDT6_7	GAGCTGTATTTGATGAAAGTA
Hs_NUDT9_5	ATGGATAATCTTATGCTAGAA
Hs_NUDT9_6	CACGCTGCAGATCCCATTATA
Hs_NUDT9_3	AAGATTAGTGCCACACTGAAA
Hs_NUDT12_6	AGCCGAGCTATTGCACATCAA
Hs_NUDT12_7	ATGATTGGTTGCTTAGCTCTA
Hs_NUDT12_5	AAGGTCGGATATTAAATAAGT
Hs_NUDT13_6	CAGGGAACGGAAGCCGTTGAA
Hs_NUDT13_5	CCACAACGTGTTGATTAACAA
Hs_NUDT13_7	TAGCCCGGATCAAGTCACTTA
Hs_NUDT14_1	CAGCGTGACCGTTCTCTTATT
Hs_NUDT14_2	AAAGGCTTGCATAGCTCCAAA
Hs_NUDT14_4	CTCCAGACAGACCATGTTCTA
Hs_NUDT15_4	CAGCAGTACTCTTCTCACTAA
Hs_NUDT15_6	CTCAAGAGCCTTTCAAGGGTA
Hs_NUDT15_7	ATCTGGTGTGATATTGTAATA
Hs_NUDT16_1	CTCCCTGTTTATATGCGTACA
Hs_NUDT16_2	CAGGTCAACACTAATACCACT
Hs_NUDT16_3	ATGTATGAAGGTGGTTCTCAA
Hs_NUDT17_7	CCCAACCATGGCAGAGGACAA
Hs_NUDT17_6	CCCGGATCCAACCAAACCCAA
Hs_NUDT17_5	TACCATCACATTGTTCTGTAT
Hs_NUDT18_1	CTGGGCCGAGATCACAGTGAT
Hs_NUDT18_2	AAGCGAGGAGTCCAAAGCTCA
Hs_NUDT18_3	ATCCTGCACCTGGTTGAACTA
Hs_NUDT19_4	TACGAAGTGAGAAGACTTGCA
Hs_NUDT19_3	CACGTTTATCCTAAGAACTCT
Hs_NUDT19_2	CACCGGATAGTGACATACCAT
Hs_DCP2_5	CCGGTGATTCATGTTTGTGAA
Hs_DCP2_6	CTGCTTATAAATGTTATTGTA
Hs_DCP2_1	CTGGGTTATCAGAGTAATGAA
Hs_NUDT21_5	CTGCACATATTACAAAGCCTA
Hs_NUDT21_1	ATCGTGATGAGAAACCTAATA
Hs_NUDT21_3	CCAAGTGTAGCTGAGCAATTA
Hs_NUDT22_4	AGGCGCCATCATCCTCTACAA
Hs_NUDT22_1	CTGGGCCTTACTTCCTACCGA
Hs_NUDT22_3	CCGAGACTTCCTGGGCACCAA
Hs_DDX58_6 (RIG-I)	AACGTTTACAACCAGAATTTA
Hs_DDX58_10 (RIG-I)	TTCTACAGATTTGCTCTACTA
Hs_DDX58_11 (RIG-I)	CTCCTCCTACCCGGCTTTAAA
Scrambled (control)	AAGGTAATTGCGCGTGCAACT

Supplementary Table 2

Proteomic analysis of bone-marrow cells drived from Nudt2$^{-/-}$ animals and Nudt2$^{+/+}$ littermate controls (WT_1-3 and KO_1-3). Three individual mice per genotype were analyzed. Table shows Protein IDs and label free quantification levels (LFQ) of individual measurements. (two-tailed Student's t-test, FDR 0.05, S0 = 0.1)

Supplementary Table 3

Nucleotide sequences used as templates in in vitro transcriptions

**Sequence Name**	**Sequence 5′ -> 3′**
**dsRNA IVT4**	TTGTAATACGACTCACTATAGGGACGCTGACCCAGAAGATCTACTAGAAATAGTAGATCTTCTGGGTCAGCGTCCC
**dsRNA IVT4_as**	GGGACGCTGACCCAGAAGATCTACTATTTCTAGTAGATCTTCTGGGTCAGCGTCCCTATAGTGAGTCGTATTACAA
**G0**	AATTCCTGCAGTAATACGACTCACTATAGGCGCCGCGGTAACGCGGCGCCACGCGGAAACGCGCC
**G0_as**	GGCGCGTTTCCGCGTGGCGCCGCGTTACCGCGGCGCCTATAGTGAGTCGTATTACTGCAGGAATT
**G8**	AATTCCTGCAGTAATACGACTCACTATAGGAACAACGGCGCCGCGGTAACGCGGCGCCACGCGGAAACGCGCC
**G8_as**	GGCGCGTTTCCGCGTGGCGCCGCGTTACCGCGGCGCCGTTGTTCCTATAGTGAGTCGTATTACTGCAGGAATT
**G4[GGAA]**	AATTCCTGCAGTAATACGACTCACTATAGGAAGGCGCCGCGGTAACGCGGCGCCACGCGGAAACGCGCC
**G4[GGAA]_as**	GGCGCGTTTCCGCGTGGCGCCGCGTTACCGCGGCGCCTTCCTATAGTGAGTCGTATTACTGCAGGAATT
**G4[GAAA]**	AATTCCTGCAGTAATACGACTCACTATAGAAAGGCGCCGCGGTAACGCGGCGCCACGCGGAAACGCGCC
**G4[GAAA]_as**	GGCGCGTTTCCGCGTGGCGCCGCGTTACCGCGGCGCCTTTCTATAGTGAGTCGTATTACTGCAGGAATT
**G4[GCAA]**	AATTCCTGCAGTAATACGACTCACTATAGCAAGGCGCCGCGGTAACGCGGCGCCACGCGGAAACGCGCC
**G4[GCAA]_as**	GGCGCGTTTCCGCGTGGCGCCGCGTTACCGCGGCGCCTTGCTATAGTGAGTCGTATTACTGCAGGAATT
**G4[GUAA]**	AATTCCTGCAGTAATACGACTCACTATAGTAAGGCGCCGCGGTAACGCGGCGCCACGCGGAAACGCGCC
**G4[GUAA]_as**	GGCGCGTTTCCGCGTGGCGCCGCGTTACCGCGGCGCCTTACTATAGTGAGTCGTATTACTGCAGGAATT
**A8**	AATTCCTGCAGTAATACGACTCACTATTAGAACAACGGCGCCGCGGTAACGCGGCGCCACGCGGAAACGCGCC
**A8_as**	GGCGCGTTTCCGCGTGGCGCCGCGTTACCGCGGCGCCGTTGTTCTAATAGTGAGTCGTATTACTGCAGGAATT
**A4[AGAA]**	AATTCCTGCAGTAATACGACTCACTATTAGAAGGCGCCGCGGTAACGCGGCGCCACGCGGAAACGCGCC
**A4[AGAA]_as**	GGCGCGTTTCCGCGTGGCGCCGCGTTACCGCGGCGCCTTCTAATAGTGAGTCGTATTACTGCAGGAATT
**A1**	AATTCCTGCAGTAATACGACTCACTATTAGGCGCCGCGGTAACGCGGCGCCACGCGGAAACGCGCC
**A1_as**	GGCGCGTTTCCGCGTGGCGCCGCGTTACCGCGGCGCCTAATAGTGAGTCGTATTACTGCAGGAATT
**T7(φ2.5)_fwd**	AATTCCTGCAGTAATACGACTCACTATAG
**T7(φ6.5)_fwd**	AATTCCTGCAGTAATACGACTCACTATTA
**Ren_IVT_PCR**	TTAAGGACGTCATTATGCTGAGTGAT

Supplementary Table 4

qRT-PCR primers used in this study

Sequence Name	Sequence 5'–> 3'
hu_NUDT2_fw	CACTGGACTCCTCCCAAAGG
hu_NUDT2_rv	TCCTCTTGGGTCTCCCTCAG
hu_NUDT12_fw	TTGGGCAAAACTTGTATCGAC
hu_NUDT12_rv	CTTCATCCTCCTATGCCAGC
hu_NUDT14_fw	AAGAGAACGGTCACGCTGTC
hu_NUDT14_rv	CCGCCTCACCCTACCTG
hu_NUDT17_fw	GACTGTCTTGCTAACCCGAAG
hu_NUDT17_rv	GGCAGGTGTAGTCCACTCTC
VSV-N_fw	ATCGGAATATTTGACCTTGTA
VSV-N_rv	ACTGTTCATTGATCATTTTGC
ms_ActB_fw	CTCTGGCTCCTAGCACCATGAAGA
ms_ActB_rv	GTAAAACGCAGCTCAGTAACAGTCCG
SFV-nsp3_fw	GCAAGAGGCAAACGAACAGA
SFV-nsp3_rev	GGGAAAAGATGAGCAAACCA
HSV-DNA Polymerase I_fw	AGCCTGTACCCCAGCATCAT
HSV-DNA Polymerase I_rv	TGGGCCTTCACGAAGAACA
Firefly Luc_fw	CTCACTGAGACTACATCAGC
Firefly Luc_rv	TCCAGATCCACAACCTTCGC
hu_GAPDH_fw	GATTCCACCCATGGCAAATTC
hu_GAPDH_fw	AGCATCGCCCCACTTGATT
Renilla Luc_fw	GAATTTGCAGCATATCTTGAACCAT
Renilla Luc_rv	GGATTTCACGAGGCCATGATAA
hu_RPLP0_fw	GGATCTGCTGCATCTGCTTG
hu_RPLP0_rv	GCGACCTGGAAGTCCAACTA
mu_TBP_fw	CCTTCACCAATGACTCCTATGAC
mu_TBP_rv	CAAGTTTACAGCCAAGATTCA
mu_Ifit3_fw	TGGTCATGTGCCGTTACAGG
mu_Ifit3_rv	GCTGCGAGGTCTTCAGACTT
mu_Il6_fw	TAGTCCTTCCTACCCCAATTTCC
mu_Il6_rv	TTGGTCCTTAGCCACTCCTTC
U1 RNA_fw	ACTTACCTGGCAGGGGAGATAC
U1 RNA_rv	ACATCCGGAGTGCAATGGATAA

Supplementary Table 5

Nucleotide guide sequences cloned in pLentiCRISPRv2

Sequence Name	Sequence 5' -> 3'
NUDT2_gRNA1	ATGAGCACCAAGCCTACCGC
NUDT2_gRNA2	AATTGCATTGTTGTCCACTT
NUDT2_gRNA3	TAACTAGGCCATGTGGAACC
NUDT2_gRNA4	CATCAGATGGCATTCATCAC
NUDT2_gRNA5	TCTCCTTGAACTGAGCCAAC
NUDT2_gRNA6	CCATTATTGAGGGGTTCAAA
XRN1_gRNA1	TTAAGAGAAGAAGTTCGATT
XRN1_gRNA2	TAAAACGCCTCCCACGCTGC
XRN1_gRNA3	GCTTGGATTAACAAGTCATG
XRN1_gRNA4	TAATGCGAAACAACACCTCC
XRN1_gRNA5	GTATCCCTGTCTCAGCGAAG
XRN1_gRNA6	TTTCACCACTTCGCTGAGAC
