# Supplementary material for: NUDT2 initiates viral RNA degradation by removal of 5′-phosphates
Source: Nat Commun. 2021 Nov 25;12:6918. doi: 10.1038/s41467-021-27239-y (PMC8616924; doi:10.1038/s41467-021-27239-y)
Supplement: Supplementary file 3 — Reporting Summary [file 41467_2021_27239_MOESM3_ESM.pdf]

nature research

Corresponding author(s): Andreas Pichlmair

Last updated by author(s): 22.10.2021

Reporting Summary

Nature Research wishes to improve the reproducibility of the work that we publish. This form provides structure for consistency and transparency in reporting. For further information on Nature Research policies, see our Editorial Policies and the Editorial Policy Checklist.

Statistics

For all statistical analyses, confirm that the following items are present in the figure legend, table legend, main text, or Methods section.

n/a	Confirmed	
☐	☒	The exact sample size (*n*) for each experimental group/condition, given as a discrete number and unit of measurement
☐	☒	A statement on whether measurements were taken from distinct samples or whether the same sample was measured repeatedly
☐	☒	The statistical test(s) used AND whether they are one- or two-sided<br>*Only common tests should be described solely by name; describe more complex techniques in the Methods section.*
☒	☐	A description of all covariates tested
☐	☒	A description of any assumptions or corrections, such as tests of normality and adjustment for multiple comparisons
☐	☒	A full description of the statistical parameters including central tendency (e.g. means) or other basic estimates (e.g. regression coefficient) AND variation (e.g. standard deviation) or associated estimates of uncertainty (e.g. confidence intervals)
☐	☒	For null hypothesis testing, the test statistic (e.g. *F*, *t*, *r*) with confidence intervals, effect sizes, degrees of freedom and *P* value noted<br>*Give P values as exact values whenever suitable.*
☒	☐	For Bayesian analysis, information on the choice of priors and Markov chain Monte Carlo settings
☒	☐	For hierarchical and complex designs, identification of the appropriate level for tests and full reporting of outcomes
☒	☐	Estimates of effect sizes (e.g. Cohen's *d*, Pearson's *r*), indicating how they were calculated

Our web collection on statistics for biologists contains articles on many of the points above.

Software and code

Policy information about availability of computer code

Data collection	IncuCyte Analysis Software (2019B Rev2)
Data analysis	GraphPad Prism (8.4.3), IncuCyte Analysis Software (2019B Rev2), Maxquant (1.6.17.0), Perseus (1.6.14.0), MAFFT (7), Pymol (2.3.2), R (4.0.2), R Studio (1.3.1056), Adobe Illustrator CS6 (16.0.3)

For manuscripts utilizing custom algorithms or software that are central to the research but not yet described in published literature, software must be made available to editors and reviewers. We strongly encourage code deposition in a community repository (e.g. GitHub). See the Nature Research guidelines for submitting code & software for further information.

Data

Policy information about availability of data

All manuscripts must include a data availability statement. This statement should provide the following information, where applicable:

- Accession codes, unique identifiers, or web links for publicly available datasets
- A list of figures that have associated raw data
- A description of any restrictions on data availability

The LC-MS/MS data and MaxQuant output generated in this study have been deposited in the ProteomeXchange Consortium via the PRIDE partner repository under accession code PXD023219 [https://www.ebi.ac.uk/pride/archive/projects/PXD023219].

April 2020

Field-specific reporting

Please select the one below that is the best fit for your research. If you are not sure, read the appropriate sections before making your selection.

☒ Life sciences ☐ Behavioural & social sciences ☐ Ecological, evolutionary & environmental sciences

For a reference copy of the document with all sections, see nature.com/documents/nr-reporting-summary-flat.pdf

Life sciences study design

All studies must disclose on these points even when the disclosure is negative.

Sample size	The sample sizes were chosen from past knowledge on the good sample size to ensure adequate power. Sample sizes are always indicated in the figure legends or related "Methods" section.
Data exclusions	No data was excluded.
Replication	At least three biological experiments were performed with similar outcomes. Fig. 2A was only conducted twice with similar results. In vivo experiments were conducted once with a sufficient number of animals to ensure adaequate statistical power in case NUDT2 would have had a significant effect.
Randomization	No randomization of data was performed due to the low sample number and the lack of influence of randomization on the experimental design and experimental approach used.
Blinding	No blinding was performed; in vitro experiments required prior knowledge for data interpretation.

Reporting for specific materials, systems and methods

We require information from authors about some types of materials, experimental systems and methods used in many studies. Here, indicate whether each material, system or method listed is relevant to your study. If you are not sure if a list item applies to your research, read the appropriate section before selecting a response.

Materials & experimental systems

n/a	Involved in the study	
☐	☒	Antibodies
☐	☒	Eukaryotic cell lines
☒	☐	Palaeontology and archaeology
☐	☒	Animals and other organisms
☒	☐	Human research participants
☒	☐	Clinical data
☒	☐	Dual use research of concern

Methods

n/a	Involved in the study	
☒	☐	ChIP-seq
☒	☐	Flow cytometry
☒	☐	MRI-based neuroimaging

Antibodies

Antibodies used	Primary antibodies used in this study were as follows: monoclonal mouse α-NUDT2 (Santa Cruz: sc-271410, 1:1000 dilution), monoclonal mouse α-β-Actin-HRP (Santa Cruz: sc-47778, 1:1000 dilution), monoclonal mouse α-myc-HRP (Roche, 11814150001, 1:2000 dilution), and secondary horseradish peroxidase (HRP)-coupled antibody rabbit α-mouse IgG (Cell Signaling, 7076, 1:2000 dilution).
Validation	All antibodies were validated by the company, information can be found under above mentioned order numbers. Moreover, anitbodies were tested in-house to validate the correct size of the identified protein. For NUDT2 we observed a decrease in band intensity in cells treated with siRNA against NUDT2.

Eukaryotic cell lines

Policy information about cell lines

Cell line source(s)	HeLa (ATCC CCL-2), HEK293T (ATCC CRL-3216), Vero E6 cells (CRL-1586) purchased from ATCC, Hep3B (obtained from Prof. R. Bartenschlager, University Hospital Heidelberg).
Authentication	Cell lines were validated by STR-profiling (Eurofins Medigenomix).
Mycoplasma contamination	All cell lines used were tested negative for mycoplasma.

Commonly misidentified lines (See ICLAC register)	No commonly misidentifed cell lines were used in this study.

Animals and other organisms

Policy information about studies involving animals; ARRIVE guidelines recommended for reporting animal research

Laboratory animals	The mice were housed and handled in accordance with good animal practice as defined by FELASA.<br>The genetically modified ES cell clone (Clone ID: EPD0146_2_H06, Cell type: JM8.N4) was injected into C57BL/6 blastocyst donors. Male chimeras were bred with C57BL/6 females to produce heterozygous Nudt2 tm1a mice. All animal experiments were approved by the responsible state office (Landesverwaltungsamt Sachsen-Anhalt), the University of Magdeburg, under permit number AZ 42502-2-1344.<br>During intranasal infections (i.n), 8–12-week-old mice were first anesthetized by i.p. injection with a mixture of ketamine (100 mg/g body weight) and xylazine (5 mg/g body weight). They then were infected with 5e6 plaque-forming units (PFU) of VSV in 20 µL PBS unless otherwise indicated. Mice that lost more than 20% of their body weight were sacrificed.
Wild animals	No wild animals were used in this study.
Field-collected samples	No Field-collected samples were used in this study.
Ethics oversight	All animal experiments were approved by the responsible state office (Referat 203, Verbraucherschutz, Veterinärangelegenheiten; Landesverwaltungsamt Sachsen-Anhalt), the University of Magdeburg, under permit number AZ 42502-2-1344.

Note that full information on the approval of the study protocol must also be provided in the manuscript.
